# PTSD recovery, spatial processing, and the val66met polymorphism

**DOI:** 10.3389/fnhum.2014.00100

**Published:** 2014-02-26

**Authors:** Jessica K. Miller, Jan M. Wiener

**Affiliations:** Department of Psychology, Bournemouth UniversityDorset, UK

**Keywords:** trauma, childhood adversity, psychology, spatial processing, navigation, hippocampus, BDNF, val66met

## Summary

Treatment for Post-Traumatic Stress Disorder (PTSD) is not always effective, and as the increasing demand for better management of PTSD and combat-related PTSD (CR-PTSD) infiltrates the UK media, so does a pressing need to understand individual variance in disease aetiology. Recent research in psychology, neuroscience and genetics has separately investigated how and why PTSD affects individuals differently. Here, we report on research on trauma, spatial processing and genetics to demonstrate that the hippocampus, part of the medial temporal lobe, is key to understanding how genes and environment interact to determine susceptibility to, and successful recovery from, PTSD. We argue that the integration of these research disciplines will bring new possibilities for prevention and treatment of PTSD within the Ministry of Defence (MOD), emergency services, National Health Service (NHS) and beyond.

## Trauma and treatment success

Individual variations within (CR-)PTSD are a challenge for PTSD research and for treatment approaches. For example, overall lifetime prevalence of exposure to traumatic events varies between 40 and 90% (Hemmings et al., 2013a) with extensive differences between individuals, military and civilian populations, and between social and occupational domains (Zaidi and Foy, 1994; Alonso et al., 2004; Evans et al., 2006; Iversen et al., 2007; Druss et al., 2009), whereas lifetime prevalence of PTSD is estimated at only 9% (Breslau et al., 2013). Further complications of under (Gee, 2013) over (Palmer, 2012) and self-reporting (Richardson et al., 2010) PTSD render its UK prevalence rate at 3% (McManus, 2009) to serve as only an estimation of the impact this condition has on individuals, communities and the wider economy.

Although the causes of variation in (CR-)PTSD prevalence and treatment success remain widely unknown (Acheson et al., 2012), age at which individuals are exposed to trauma is known to influence PTSD aetiology: both in terms of early life stress (Brewin et al., 2000; Vasterling and Brewin, 2005; McGowan and Szyf, 2010) and the development of skills required to both verbalize and spatially contextualize trauma (van der Kolk, 2003; Betts et al., 2012); and with regard to dementia (Duax et al., 2013).

With the spectrum of treatment (Lindauer et al., 2005) and its success (Gaskell and British Pyshcological Society, 2005) being broad and the UK military charity Combat Stress delivering PTSD treatment on the principal^1^ that over one third of veterans will not recover well, there is an increasing and genuine demand for efficient assessment, referrals and treatment in a very diverse PTSD population. We propose that understanding individual variance in hippocampal integrity will provide a stable and objective means of quantifying PTSD susceptibility and treatment success.

## PTSD, the hippocampus, and spatial processing

PTSD has been associated with hippocampal integrity and volume (Gilbertson et al., 2002; Apfel et al., 2011), with chronic PTSD in veterans being associated with a 6% reduction in hippocampal volume compared to recovered veterans (Gilbertson et al., 2002; Apfel et al., 2011). Moreover, it is understood that:

1. Hippocampal integrity is affected by stress (Sapolsky, 2000; Vasterling and Brewin, 2005; Wang et al., 2010);
2. Stress from trauma exposure poses a real threat to hippocampal functionality (Brewin et al., 2010; Acheson et al., 2012; Pitman et al., 2012); and
3. PTSD symptoms (such as management of intrusions, inadequate integration of sensory memories in recall, lack of “self-referential perspective” and fear contextualization) are related to hippocampal activity (Jeansok and Fanselow, 1992; Philips and Le Doux, 1992; Ehlers and Clark, 2000; Astur et al., 2006; Bisby et al., 2010; Brewin et al., 2010; Acheson et al., 2012; Pitman et al., 2012).

While there is growing evidence for the importance of hippocampal integrity for PTSD resilience and recovery, the question *how to best assess it* remains. Hippocampal function can be measured in many ways, including pattern separation (Clelland et al., 2009) and context-dependent fear conditioning (Gerlai, 2001; Ji and Maren, 2008). We suggest hippocampal-dependent spatial processing (King et al., 2002; Bird and Burgess, 2008; Bisby et al., 2010) is particularly useful for trauma research. Spatial processing abilities are known to be negatively affected by trauma (Gilbertson et al., 2007; Bisby et al., 2010; Tempesta et al., 2012, Smith et al., manuscript in preparation), are highly relevant for those occupations which demand navigation competence and simultaneously elevate risk of trauma exposure (such as the Armed Forces and emergency services), and can be objectively quantified (Bird and Burgess, 2008).

The hippocampus has been implicated in allocentric processing, a specific type of spatial processing which involves viewpoint-independent manipulations of spatial relations between locations (Burgess et al., 2002; Burgess, 2006). Allocentric processing allows for the use of “observer” or “field” perspectives in trauma processing, and this dates back to Freudian psychoanalysis of anxiety-provoking memories (McIsaac and Eich, 2004; Eich et al., 2011, 2012). Explicit references to allocentric (or indeed egocentric) processing are unlikely to be found in trauma literature simply because this terminology is more familiar to the domain of spatial cognition. An example of therapeutic field using similar constructs is offered by Ehlers and Clark (2000) who employ the term “the self-referential,” in their theoretical model of PTSD. Neuropsychologists interested in spatial processing might refer to such self-referential processing as “egocentric” (or non-allocentric). Nonetheless using perspective (and indeed spatial perspective) in contextualizing evocative and sensory trauma is referred to in trauma literature (Steel et al., 2005; Neuner et al., 2008), and a specific example of this is offered with Bisby and colleagues' recent non-clinical samples (Bisby et al., 2010). Forthcoming findings from research into the effect of PTSD on configural memory may substantiate this relationship between allocentric processing and trauma processing further (Smith et al., manuscript in preparation). Brewin goes so far as to suggest that future clinical interventions for PTSD should involve further attempts to change information processing biases in Vasterling and Brewin (2005), and this paper adopts spatial cognition terminology (i.e., “allocentric processing”) to shed more light on role of the hippocampus in processing traumatic and spatial information.

## Hippocampal integrity

We have argued that trauma and PTSD affect hippocampal integrity (Acheson et al., 2012) and that this integrity is important for success in some treatments (Neuner et al., 2008; Bisby et al., 2010; Adenauer et al., 2011). This poses a dilemma: how can the hippocampus be appropriately employed to process the trauma, if trauma is disrupting its own function?

The answer may lie in the fact that the hippocampus is able to generate neurons throughout life (Eriksson et al., 1998; Andersen et al., 2007). It can increase in volume through spatial training (Maguire et al., 2000) and also increase in density through meta-cognition (Holzel et al., 2010). Moreover, spatial training procedures which force participants to adopt allocentric, viewpoint-independent perspectives have produced regenerative effects in the hippocampus (Whitlock et al., 2006; Lövdén et al., 2011).

These findings strongly suggest that hippocampal integrity and function can be improved using training procedures that employ spatial tasks. While this already has potential implications for PTSD recovery—particularly for interventions that make use of spatial contextualization— recent results from genetics research suggest that training success may depend on specific genotypes (Lövdén et al., 2011).

## DNA

Many genes have been associated with PTSD symptomology (Koenen et al., 2009; Schmidt et al., 2011; Skelton et al., 2012)—several by means of the phenotype or “candidate approach” (Yehuda et al., 2011; Skelton et al., 2012) which selects genes already known to result in similar traits as the PTSD symptom of interest (Gottesman and Gould, 2003; Acheson et al., 2012). Whilst this has provided insight into many areas of the neurobiological system and various “symptoms” associated with PTSD, Hemmings et al. (2013b) recently stated that “no gene variant has yet been reported as unequivocally involved in the development of this disorder [PTSD].” We suggest that the Brain Derived Neurotropic Factor (BDNF) gene may be that gene: primarily because of its role in hippocampal processing and its recent associations with PTSD.

BDNF determines levels of N-acetylaspartate (NAA) in the hippocampus, which is a putative marker of neural integrity (Egan et al., 2003; Salehi et al., 2013), is crucial for maintaining a healthy hippocampal volume (Carballedo et al., 2013) and plays an important role in managing the stress response (Suliman et al., 2013). The BDNF vall66met polymorphism involves three genotypes: val/val, val/met and met/met. In the Caucasian population, 70% of the population carry the single nucleotide polymorphism val66val, 27% carry val66met and 3% carry met66met (Petryshen et al., 2010).

## What evidence links BDNF to PTSD?

A wide literature introduces the role of BDNF and val66met in psychological wellbeing, in overall development (Casey et al., 2009), in mood disorders (Duman and Monteggia, 2006), depression (Aguilera et al., 2009; Gatt et al., 2009) and even attempted suicide (Perroud et al., 2008; Pregelj et al., 2011).

The BDNF polymorphism has been associated with childhood trauma, with carriers of the “met” variation being particularly sensitive to the impact of child abuse and recent stress (Elzinga et al., 2011). BNDF variations are also considered as modifiers of the risk of childhood trauma in obsessive-compulsive disorder (Hemmings et al., 2013a,b; Suliman et al., 2013) and as mediators of the impact of childhood adversity on lifetime depression (Carver et al., 2011). A plethora of studies demonstrate a connection between the val66met polymorphism of BDNF and PTSD in relation to: extinguishing the fear and startle response (Rattiner et al., 2004; Zhang et al., 2014); PTSD symptomology and severity (Koenen et al., 2009; Frielingsdorf et al., 2010; Hemmings et al., 2013b); psychotic PTSD (Pivac et al., 2012); and the efficacy of PTSD therapy (Felmingham et al., 2013).

Recently, Zhang et al. (2014) reported that amongst a sample of 461 trauma exposed US soldiers deployed in Afghanistan and Iran, 10% had probable PTSD (Zhang et al., 2014). Within that group (*n* = 42), the frequency of met/met genotypes was nearly three-fold higher than in the controls, and the frequency of val/met genotypes was two-fold higher in individuals with probable-PTSD than in controls. The frequency of the BDNF val66met genotypes was significantly higher in those with PTSD and in those with exaggerated startle (a core symptom of PTSD) than in non-PTSD groups. Overall, the val66val genotype has been suggested to increase PTSD resilience, while the val66met allele increases vulnerability of PTSD (Koenen et al., 2009; Elzinga et al., 2011; Hemmings et al., 2013b; Zhang et al., 2014).

## What is the relation between the BDNF polymorphism and the hippocampus?

“Met” carriers develop smaller hippocampi (Szeszko et al., 2005), especially if they are exposed to early life stress (Gatt et al., 2009)—and as they age, are more likely to show lower hippocampal activity and resilience (Raz and Rodrigue, 2006; Fehér et al., 2013; Wiener et al., 2013) poorer performance on spatial tasks (Sanchez et al., 2011) and are less prone to explore unfamiliar environments (Chen et al., 2006). Furthermore, val66met has been shown to impair the hippocampal plasticity induced by SSRI anti-depressants (such as fluoxetine) which are often used in the treatment of PTSD (Bath et al., 2012). Kleim et al. (2006) showed that changes in neural plasticity and motor function are mediated by the val66met BDNF polymorphism, and Lövdén et al. (2011) demonstrated that increased levels of hippocampal NAA (a putative marker of neural integrity) as a result of spatial training were restricted to BDNF val homozygotes (val/val). Val/met heterozygotes and met/met homozygotes did not benefit from the spatial training which required allocentric processing, the very processing which is thought to be so useful to manage trauma.

## Can we predict success rates of different PTSD treatments?

We have reviewed research demonstrating that:

1. PTSD is inextricably linked to the hippocampus (Astur et al., 2006; Bisby et al., 2010; Brewin et al., 2010; Acheson et al., 2012; Pitman et al., 2012);
2. Hippocampal integrity and development has a strong genetic component (Szeszko et al., 2005; Gatt et al., 2009; Lövdén et al., 2011) and
3. Some forms of PTSD treatments rely on hippocampal processing (McIsaac and Eich, 2004; Vasterling and Brewin, 2005; Adenauer et al., 2011).

**Figure 1:**
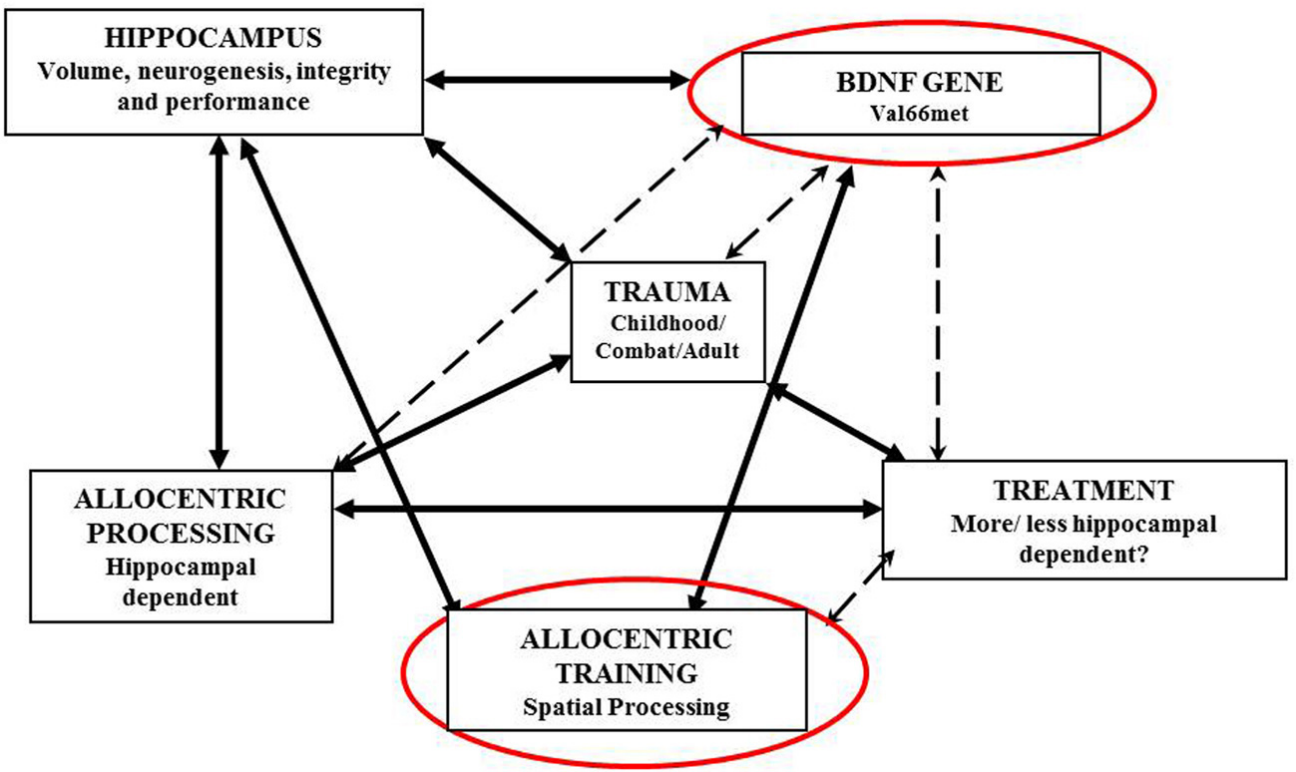
**From DNA to treatment success: BDNF, the hippocampus, spatial, and trauma processing**.

Figure 1 illustrates the interrelations between BDNF, the hippocampus, spatial processing and trauma processing.

Together, these findings allow for an intriguing conclusion: the success rate of specific PTSD treatments may well be predicted by analysing patients' BDNF genotype. Specifically, we argue that PTSD therapies involving spatial contextualization of traumatic event (such as exposure therapy) will have lower success rates in val/met heterozygotes and met/met homozygotes than in val/val homozygotes, especially if individuals have been exposed to early life stress. This is because spatial contextualization is dependent on hippocampal processing, and hippocampal integrity and plasticity is mediated by the val66met BDNF polymorphism.

In conclusion, we suggest that genetic analysis can help to predict the success of different types of PTSD treatments and methods of trauma processing, and may be used to improve referral pathways and eventually PTSD recovery rates.

Research between UCL, Bournemouth University, the NHS and Combat Stress is currently being undertaken to quantify the relationship between the BDNF gene, combat and childhood trauma processing and hippocampal-dependent navigation, with the intention of providing new insight into the experience of PTSD in the UK.

## Footnotes

^1^Based on the model used by the Australian Veterans Rehabilitation programme (Combat Stress Annual Report 2011–12).
